# Self-induced vomiting and dental erosion – a clinical study

**DOI:** 10.1186/1472-6831-14-92

**Published:** 2014-07-29

**Authors:** Marte-Mari Uhlen, Anne Bjørg Tveit, Kjersti Refsholt Stenhagen, Aida Mulic

**Affiliations:** 1 Department of Cariology, Institute of Clinical Dentistry, Faculty of Dentistry, University of Oslo, PO Box 1109, Oslo N-0317, Norway

**Keywords:** Dental erosion, Eating disorders, Vomiting

## Abstract

**Background:**

In individuals suffering from eating disorders (ED) characterized by vomiting (e.g. bulimia nervosa), the gastric juice regularly reaches the oral cavity, causing a possible risk of dental erosion. This study aimed to assess the occurrence, distribution and severity of dental erosions in a group of Norwegian patients experiencing self-induced vomiting (SIV).

**Methods:**

The individuals included in the study were all undergoing treatment at clinics for eating disorders and were referred to a university dental clinic for examinations. One calibrated clinician registered erosions using the Visual Erosion Dental Examination (VEDE) system.

**Results:**

Of 72 referred patients, 66 (63 females and three males, mean age 27.7 years) were or had been experiencing SIV (mean duration 10.6 years; range: 3 – 32 years), and were therefore included in the study. Dental erosions were found in 46 individuals (69.7%), 19 had enamel lesions only, while 27 had both enamel and dentine lesions. Ten or more teeth were affected in 26.1% of those with erosions, and 9% had ≥10 teeth with dentine lesions. Of the erosions, 41.6% were found on palatal/lingual surfaces, 36.6% on occlusal surfaces and 21.8% on buccal surfaces. Dentine lesions were most often found on lower first molars, while upper central incisors showed enamel lesions most frequently. The majority of the erosive lesions (48.6%) were found in those with the longest illness period, and 71.7% of the lesions extending into dentine were also found in this group. However, despite suffering from SIV for up to 32 years, 30.3% of the individuals showed no lesions.

**Conclusions:**

Dental erosion commonly affects individuals with ED experiencing SIV, and is more often found on the palatal/lingual surfaces than on the buccal in these individuals, confirming a common clinical assumption.

## Background

Eating disorders (ED) are conditions characterized by restricted food intake or binge eating, and often by self-induced vomiting (SIV). In addition to having the potential to impair both physical health and psychosocial functioning [1], these disorders could also have an impact on oral health. Among oral complications is dental erosion, an irreversible loss of tooth substance as a consequence of exposure to acids that do not involve bacteria [2]. Such acids may enter the oral cavity from extrinsic (e.g. acidic foodstuff), as well as from intrinsic sources (gastric acid).

The prevalence of ED has been studied in different countries. A nationally representative survey from the U.S. estimated that the lifetime prevalence of ED ranged from 0.6 – 4.5% among adults [3] and from 0.3 – 0.9% among adolescents [4]. According to questionnaire-based, epidemiological studies in Norway, the lifetime prevalence of any ED among adolescents was found to be 12.5% [5], and of anorexia nervosa (AN) and bulimia nervosa (BN) among adult females 0.4% and 1.6%, respectively [6]. A recent study in Finland reported that the lifetime prevalence of AN among young adults was 1.3% and of BN 1.1% [7], whereas a large German study found the prevalence of AN and BN among children and adolescents to be 0.14% and 0.11%, respectively [8].

Significant higher values of erosive tooth wear have been found in patients suffering from ED compared to control groups [9-15]. Johansson et al. [15] found that patients with ED had an 8.5-times increased risk of having dental erosion compared to healthy controls, and that individuals with a longer history of ED had lesions more frequently.

An obvious risk factor considering dental health in individuals suffering from BN, certain forms of AN or other EDs, is repeated vomiting. The stomach content, which can have a pH close to 1 [16,17], repeatedly reach the mouth and may be destructive to the tooth substance [11]. Recent studies have shown more dental erosive wear among adolescents reporting vomiting [15,18], and that these individuals have a 5.5-times higher risk of dental erosions than those without such behaviour [15].

As emphasized by Hellstrom [19], eating disorders are serious and potentially fatal conditions, where the implications on dental health are reasonably not considered among the most important. However, although many of the physical manifestations of these disorders are reversible, those affecting the teeth’s hard tissue are not [20,21]. Dentists play a significant role in identifying, preventing and treating dental erosion, and it is therefore important to be aware of risk indicators associated with this condition. A common apprehension among clinicians is that dental erosions are typically localized on the palatal surfaces of the upper front teeth in patients with vomiting or regurgitation, while erosions on the buccal or facial surfaces may be a result from high consumption of highly acidic foods and drinks. Although there is some support for this in the literature, [9,10,17,19,22-25], several of these studies have limitations. Three of the studies have a low number of participants included [19,22,24], and calibration of examiners prior to onset of the studies was performed in only one study [23]. In addition, two of the studies did not distinguish between enamel and dentine lesions, making the severity assessment of the erosions difficult [10,25]. This complicates the examiners’ ability to monitor the progression of the lesions.

It is often assumed by clinicians and researchers that ED patients have an increased risk of developing dental erosion. However, more information about dental erosions in this risk group is required in order to adequately prevent and treat them. Therefore, the aim of this study was to assess the occurrence, distribution and severity of dental erosions in patients suffering from eating disorders characterized by self-induced vomiting, and the possible association between dental erosions and the duration of the eating disorder.

## Methods

### Participants

The individuals included in the study were all undergoing psychiatric and/or medical outpatient treatment at clinics for eating disorders during 2005 – 2013. Because of the assumed relation between eating disorders and dental problems, all patients at these clinics were offered a referral for a dental examination. The patients who were interested were then offered a dental examination at a university dental clinic (University of Oslo, Norway). The eating disorder diagnoses were made by the professional team at the eating disorder clinics. Of 72 referred patients examined at the university dental clinic, 62 had been diagnosed with BN, eight with AN, one with binge-eating disorder (BED) and one with an unspecific eating disorder. Self-induced vomiting was, or had been, a part of the eating disorder for 67 patients, and only these individuals were further studied. After the examination, one additional individual was excluded from the study because of crowns and onlays on all lower molars and upper front teeth.

### Interview

Prior to the dental examination, each patient was interviewed by one examiner. The standardized interview was based on questions from a previously tested questionnaire [26,27], and discussed the patient’s present medical condition, other diseases/diagnoses and medical history. In addition, the examiner asked each patient about their dietary habits, such as consumption of acidic beverages and foods. This consumption was assessed by frequency questions with five possible responses: several times daily, once daily, 3–5 times weekly, 1–2 times weekly and less than once weekly. The participants were also asked if they vomited after eating, and if so, how often (daily, several times weekly, monthly and occasionally) and how long time since the last episode of vomiting.

The duration of self-induced vomiting was recorded during the interview, with three possible responses: 3–7 years, 8–10 years and more than 10 years duration of self-induced vomiting. Only a few participants specified the time of last episode of vomiting, and because this ranged from weeks to years, it was therefore not further considered in the study. The frequency of SIV was registered as times of vomiting per week, and ranged from two to 210 times per week.

### Calibration and clinical examination

The intra-oral clinical examination was performed by one previously calibrated clinician (AM). The examiner was calibrated with four other clinicians (intra- and inter-examiner agreement values of mean κ_w_ = 0.95 and mean κ_w_ = 0.73; range 0.71 – 0.76, respectively). For more details see Mulic et al. [28].

The clinical examination was performed in a dental clinic with standard lighting, using mirrors and probes. Access saliva was removed from the teeth with compressed air and cotton rolls. The lingual/palatal and buccal surfaces of all teeth, and the occlusal surface of premolars and molars, were examined. For severity grading of dental erosion, a well established scoring system with the ability to diagnose early stages, as well as more advanced stages of erosion, was required. Scoring of dental erosion was therefore performed according to the Visual Erosion Dental Examination (VEDE) system [27-29]: Score 0: No erosion; score 1: Initial loss of enamel, no dentine exposed; score 2: Pronounced loss of enamel, no dentine exposed; score 3: Exposure of dentine, < 1/3 of the surface involved; score 4: 1/3-2/3 of the dentine exposed; score 5: > 2/3 of dentine exposed. The number and distribution of affected teeth and surfaces were also registered. When surfaces were either filled, repaired with a crown or a veneer, affected by attrition or abrasion, or the tooth had been extracted, the surfaces and teeth were recorded as missing and excluded.

### Ethical considerations

The study was approved by the local Regional Committee for Medical Research Ethics and The Norwegian Social Science Data Services. Written, informed consent was obtained from all participants.

### Statistical analysis

The statistical analyses were performed using the Statistical Package for the Social Sciences (SPSS, Chicago, IL, USA, version 20). Presence of dental erosive wear was used as the dependent variable. Frequency distributions, descriptive and bivariate analyses (Chi-square test) were conducted to provide summary statistics and preliminary assessment of the associations between independent variables and the outcome.

The level of significance was set at 5%.

## Results

### Participants

Of the 66 individuals included, 63 were female and three were male, with a mean age of 27.7 years (range: 20 – 48). Two participants did not answer the question regarding the duration of their eating disorder, but for the remaining 64 participants, the mean duration of the disorder was 10.6 years (range: 3 – 32 years).

### Prevalence, severity and distribution of dental erosions

Of the 66 individuals included in the study, 20 (30.3%) showed no signs of dental erosions. The mean age of these individuals was 27.7 years (range: 20 – 48) and the duration of ED ranged from 3 to 32 years (mean: 10.6). Of the 46 individuals with dental erosion, only 43 answered the question of the duration of SIV.

Dental erosions were found in 46 individuals (69.7%), 43 women and three men. Of these, 19 individuals had enamel lesions only, while 27 had both enamel and dentine lesions. Of the individuals with erosions, 35 participants (76.1%) had five or more teeth affected, while 12 (26.1%) had 10 or more teeth with erosive lesions. Four individuals (9%) had ≥10 teeth with dentine lesions. Erosions grade 4 or 5 (severe erosion) were found in only six of the individuals, three of these had been suffering from ED for 16, 21 and 28 years, while two had been diagnosed six and nine years ago.

Dentine lesions appeared most frequently on occlusal surfaces (n = 66, 58.4%), followed by palatal surfaces on front teeth (n = 22, 19.5%). Of the 66 surfaces with dentine erosions on occlusal surfaces (Table 1), lower first molars had most often dentine involvement (n = 28), while enamel lesions were most frequently found on upper central incisors (n = 58).

**Table 1:** Distribution and severity of affected tooth surfaces according to duration of SIV

**Affected tooth surfaces**	**Duration of SIV 3–7 years**			**Duration of SIV 8–10 years**			**Duration of SIV >10 years**			**All individuals**		
	**(n = 14)**			**(n = 14)**			**(n = 16)**			**(n = 44)**		
	**E**	**D**	**E + D**	**E**	**D**	**E + D**	**E**	**D**	**E + D**	**E**	**D**	**E + D**
**Front B (n = 79)**	21	0	21	26	2	28	30	0	30	77	2	79
**Front P (n = 147)**	47	2	49	31	4	35	47	16	63	125	22	147
**Lateral segments B (n = 15)**	0	0	0	0	0	0	11	4	15	11	4	15
**Lateral segments P (n = 33)**	0	0	0	8	0	8	6	19	25	14	19	33
**Lateral segments O (n = 158)**	23	15	38	34	9	43	35	42	77	92	66	158
**All surfaces**	91	17	108	99	15	114	129	81	210	319	113	432

E = Enamel; D = Dentine; Front B = Buccal surfaces of canines and incisors in both upper and lower jaw; Front P = Palatal surfaces of canines and incisors in upper jaw; Lateral segments B = Buccal surfaces of premolars and molars in both upper and lower jaw; Lateral segments P = Palatal surfaces of premolars and molars in upper jaw; Lateral segments O = Occlusal surfaces of premolars and molars in both upper and lower jaw.

### Duration of SIV and dental erosions

**Figure 1:**
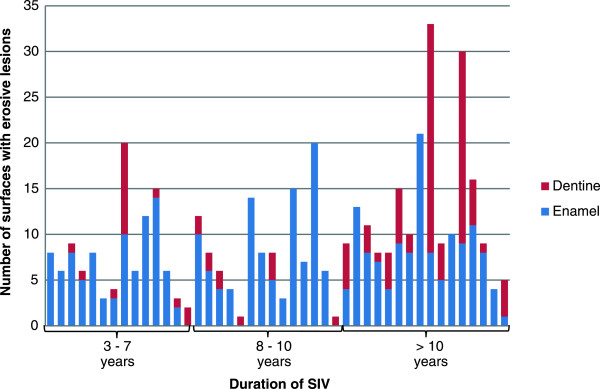
Number of affected surfaces with erosive lesions in relation to duration of SIV.

The group of individuals (n = 16) with the longest duration of SIV (>10 years) had 71.7% of the dentine lesions and 40.4% of the enamel lesions (Table 1).Of the individuals included in the study, eight (17.4%) had five or more teeth with dentine lesions. Only seven of these individuals answered the question of the duration of SIV, and for those who did, the mean duration was 17.1 years (range: 6 – 28). Ten participants (21.7%) had five or less surfaces with erosions (Figure 1). Seven of the individuals had lesions on occlusal surfaces only. Eight of those with five or less involved tooth surfaces had been suffering from ED for 5 – 10 years, while two patients reported duration of 26 and 28 years, respectively.

The distribution of the total number of surfaces with erosive lesions (n = 432) according to the duration of SIV is presented in Table 1. The palatal surfaces were most frequently affected (41.6%), followed by occlusal surfaces (36.6%), regardless of the duration of SIV (Table 1). However, the longer the duration of SIV, the more lesions were recorded on the palatal surfaces of the front teeth and in the lateral segments (Table 1). The same was found on the buccal surfaces; though the prevalence was lower (Table 1).

The individuals who had been suffering from ED for more than 10 years had significantly more buccal lesions in the lateral segments than those with a shorter duration of the disorder (p = 0.02). This group did also have significantly more palatal lesions in the lateral segments than those who had been suffering from ED for 3 – 7 years (p < 0.01) and 8 – 10 years (p = 0.04). Nine individuals who had induced vomiting for more than 10 years showed no signs of dental erosions, and in two individuals less than five teeth were affected.

### Consumption of acidic beverages

Of the 54 individuals who answered the questionnaire, 24 reported a high daily intake of acidic beverages (≥0.5 liters per day), 30 participants reported a low consumption (<0.5 liters per day), and of these 17 (70.8%) and 23 persons (76.7%) showed dental erosions, respectively. The mean age in these groups was 28.4 (range: 21 – 48) and 27.5 years (range 20 – 43), and the mean duration of SIV was 12.0 (range: 3 – 28) and 9.9 years (range: 3 – 21). The mean number of teeth with erosions was 4.3 in the high consumption group and 5.8 in the low consumption group. Only one person in the high consumption group showed erosions grade 4 and 5, and no more than 9 teeth were affected, while in the low consumption group 3 individuals showed erosions grade 4 or 5, and six participants had 10 or more teeth affected with dental erosions.

## Discussion

In this study, dental erosions were found in 69.7% of the individuals having a history of self-induced vomiting (SIV). This is in the lower range of previously reported prevalence (47 – 93%) among bulimic patients [11,12,30]. Although all the individuals included in the current study had a history of SIV, and thereby were at risk of developing dental erosions, 30.3% of the participants did not display any signs of erosion lesions. Previous studies have reported different findings. All patients with ED in the study by Robb et al. [10] had significantly more abnormal tooth wear (erosion) than the healthy control group, this finding being most prominent in the SIV group. None of the 23 women with AN in the study by Shaughnessy et al. [31] showed dental erosions, even though 26% of the participants reported a history of binge-eating/purging activity. Rytomaa et al. [11] found that 13 of 35 bulimics did not suffer from dental erosions. The observation that not all bulimic patients show a pathological level of tooth wear has also been reported by Milosevic and Slade [9] and Touyz et al. [30]. Although vomiting has been related to the occurrence of erosive wear, the study by Robb et al. [10] showed that those who suffered from AN, but did not vomit, also showed more erosions than the control population.

Dental erosions can be caused by acids from extrinsic (e.g. acidic foodstuff) as well as from intrinsic sources (gastric acid). In the present study, one of the inclusion criteria was self-induced vomiting, a challenge to the enamel due to exposure to gastric acid. Nearly half (n = 24) of the participants who completed the questionnaire also reported a high daily intake of acidic beverages. It is likely that individuals who induce vomiting up to several times per day have a higher risk of developing dental erosion than those who never, or more seldom, practice this behaviour. It is reasonable to assume that individuals who, in addition to exposing their teeth to gastric acids several times per day, often consume acidic beverages, have a greater risk of developing dental erosion than those who do not. However, in the present study, there were more erosions and more severe lesions in the group with low consumption of acidic beverages than in the group with high consumption. Bartlett and Coward [16] compared the erosive potential of gastric juice and a carbonated beverage in vitro and found that gastric juice had greater potential to cause dental erosions in enamel and dentine than a carbonated drink. The authors pointed out that the result reflects the lower pH and titratable acidity of gastric juice. This could be a reason why more lesions were not found in those individuals who consumed large amounts of acidic beverages in addition to SIV.

For patients with ED it is difficult to evaluate the risk of various dietary factors, vomiting and/or unfavourable saliva factors. Information about the frequency and duration of SIV is associated with uncertainties, because ED often are associated with shame and denial. It is often a general finding that these individuals are well-educated and well-informed about the condition. Many of them normally choose healthy diets devoid of sweets and sugary soft drinks. In contrast, when they have episodes of binge-eating they select “junk food”, which is high in fat, sugar, salt and calories [12].

The results from the present study showed that the participants who had been practicing SIV for more than 10 years showed more erosions and more severe lesions (with exposed dentine). Frequent acid exposures may have a detrimental effect on the teeth’s hard tissue, and particularly if the exposures continue over a long period of time. This finding is in accordance with results from Johansson et al. [15] and Altshuler et al. [25], who found a significant association between the duration of the ED and the prevalence of dental erosions. In addition, Dynesen et al. [14] showed that the duration of the ED had a significant influence on the severity grade of the erosive lesions. However, other studies did not find any association between frequency, duration of vomiting and dental erosion [9,10]. In the present study nine individuals who had induced vomiting for more than 10 years showed surprisingly no signs of dental erosions, and in two individuals less than five teeth were affected.

The different results from the studies mentioned, and the fact that one third of the individuals in the present study did not show any erosive lesions despite regular vomiting, might be explained by individual differences in the susceptibility to erosion. It is still not clear what factors are relevant for the development and progression of erosion in these patients. Saliva factors, salivary flow rate, the pellicle and the composition of the enamel may be as important as the frequency of acid exposures [10].

It has often been speculated that differences in the composition of saliva could be responsible for the rapidly progressing erosive substance loss in patients with vomiting-associated ED [32]. A lower salivary pH in ED patients than in healthy controls has been documented by Touyz et al. [30], but in contrast Milosevic et al. [33] did not find any differences between BN patients and controls. Schlueter et al. [34] suggested that enhanced proteolytic activity in the saliva of bulimic patients might contribute to an altered buffering capacity of the saliva, as well as development and progression of dental erosion through degradation of dentine and the pellicle. Levels of amylase, immunoglobulin and electrolytes have also been investigated, but the findings differ substantially [32]. Several studies have shown a significantly lower unstimulated salivary flow in bulimic patients than in controls [11,14,19]. Many ED patients are prescribed antidepressants or other psychopharmaceutical medication, that are known to reduce salivary flow, and Dynesen et al. [14] showed that xerogenic medication significantly lowered unstimulated flow rate in this patient group.

The assumption that dental erosions caused by vomiting or regurgitations are typically localized on the palatal surfaces of the upper front teeth, and that erosions caused by high consumption of acidic foods and drinks are found on buccal surfaces, has led to efforts to relate the location of erosive lesions to the etiology of the condition [17,22,23]. From a clinical point of view, it is important to investigate whether it is possible to differentiate between erosions caused by SIV and erosions caused by consumption of acidic foodstuff. Hellstrom [19] reported that while lingual erosions were a frequent finding in individuals experiencing SIV, such lesions did not appear in individuals without this behaviour. Lussi et al. [23] found that chronic vomiting appeared to be the variable most indicative for lingual erosions. The present results showed that the majority of the lesions were found on the palatal surfaces and that the individuals with the longest duration of SIV had significantly more buccal and palatal lesions in the lateral segments than those with a shorter duration of the disorder. The more severe lesions (with exposed dentine) appeared most frequently on the occlusal surfaces of the lower first molars, followed by the palatal surfaces on the upper incisors. These results were consistent with work previously reported by Mulic et al. [18] in a study of healthy adolescents, and can partly be explained by the position of these teeth in the mouth and partly by their early time of eruption. The lower first molars are the first permanent teeth to erupt, they have an important function concerning occlusion and chewing, and acidic liquids naturally gravitate towards the floor of the mouth. The upper incisors also erupt at an early age, and as the rough surface of the tongue serves as a short-time reservoir for acids from foodstuff and liquids, the movements of the tongue have the potential to cause both an abrasive and an erosive effect (perimylolysis) on the palatal surface of the upper front teeth [35]. The rinsing effect of saliva is weaker on the upper teeth than on the lower, and the saliva clearance and buffer capacity are even lower in individuals with reduced saliva secretion [17].

Vigorously and frequent tooth brushing is a common habit in ED patients, and in combination with the repeated exposure to stomach acid, this could also contribute to the increased erosive tooth wear in this group [15,19]. However, a study concerning oral hygiene habits did not ascertain why some of the vomiting bulimic patients suffered from severe erosions and others did not [36].

As expected, the prevalence of dental erosion in the individuals experiencing SIV was high. In addition, the duration of SIV seemed to influence the number of palatal and buccal lesions.

However, the present study does have some limitations: The proportion of male participants versus females was small. This, however, reflects the gender distribution shown in the study by Jaite et al. [8], where as much as 92.7% of individuals with bulimia nervosa were female, and seems to reflect the population prevalence of ED. Information on e.g. the exact duration of disease, frequency of SIV, oral hygiene habits and eating habits is unfortunately difficult to obtain, as it is based exclusively on the participants’ subjective memory and their willingness to share. One might consider using standardized questionnaires instead of interviews, but these have approximately the same limitations in this matter. Another possible limitation of this study is that the participants were all undergoing treatment at clinics for eating disorders. Thus, the study does not include individuals who do not receive treatment for their disease, and the results might have been different if corresponding information from such individuals was obtained. Saliva secretion was not measured in this study. Considering that one third of the participants with EDs in a study by Rytomaa et al. [11] had decreased unstimulated saliva secretion, and hence decreased protection against dental erosion, this might have been an interesting addition to the study. However, Dynesen et al. [14] found that although individuals with vomiting had a significantly lower unstimulated salivary flow rate compared to a control group, this did not significantly influence dental erosion.

## Conclusion

In the present study, dental erosions frequently affected individuals with ED experiencing SIV, and erosions were more often found on the palatal than on the buccal surfaces in these individuals, supporting a common clinical assumption. However, an interesting finding was that nearly one third of the individuals who had been experiencing SIV presented no visual signs of dental erosion. This emphasizes the necessity of further investigation concerning the etiology of the condition, and of communicating the increasing knowledge of ED and dental erosion to clinical practitioners. Since dentists often are the first health care professionals to whom persons with previously undiagnosed ED may present [32], and as dental care is an important part of the overall treatment for these patients, it is of imperative importance that dentists have an adequate knowledge regarding such disorders and how to prevent and treat the resulting oral consequences.

## Competing interests

The authors declare that they have no competing interest. The authors alone are responsible for the content and writing of the paper.

## Authors’ contributions

MMU and AM carried out the data collection; MMU did the data analysis and writing of the article. ABT initiated the idea and along with KRS and AM supervised the project and assisted in writing/editing of the article. All authors have read and approved the final manuscript.

## Pre-publication history

The pre-publication history for this paper can be accessed here:

http://www.biomedcentral.com/1472-6831/14/92/prepub
